# Urease-null soybean (*eu3-a*) under salt and copper stress: nitrogen metabolism, antioxidant defense, and arginine pathway genes

**DOI:** 10.1007/s00425-026-05001-2

**Published:** 2026-04-16

**Authors:** Sarah Caroline R. de Souza, Neidiquele M. Silveira, Vanessa R. Tofanello, Joe Carmine Polacco, Paulo Mazzafera

**Affiliations:** 1 Department of Plant Biology, Institute of Biology, University of Campinas, PO Box 6109, Campinas, SP 13083-970 Brazil; 2 Department of Botany, Federal University of São Carlos, PO Box 676, São Carlos, SP 13565-905 Brazil; 3 Department of Biodiversity, Institute of Biosciences, São Paulo State University (UNESP), PO Box 1515, Rio Claro, SP 13506-900 Brazil; 4 Department of Biochemistry, Interdisciplinary Plant Group, University of Missouri, 117 Schweitzer Hall, Columbia, MO 65211 USA

**Keywords:** Arginase, Nitrate, Polyamines, Proline, Tryptophan, Urea

## Abstract

**Main conclusion:**

**The** ***eu3-a*** **mutant exhibited greater tolerance to salt stress but increased sensitivity to copper stress, with distinct impacts on nitrogen metabolism, photosynthesis, and antioxidant responses.**

**Abstract:**

The *eu3-a* soybean mutant is urease-null, lacking all urease activity responsible for catalyzing the hydrolysis of urea into ammonia and carbon dioxide. In this study, the urease-null *eu3-a* soybean mutant was used to assess the saline and copper stresses on nitrogen metabolism. Seeds of *eu3-a/eu3-*a and the corresponding dominant homozygous *Eu3* precursor line were collectively referred to as near-isogenic lines (NILs). Experiments were conducted under hydroponic conditions using plants at the reproductive stage (R1–R2) and subjected to either salinity stress (NaCl: 0, 50, and 100 mM) or copper stress (CuCl_2_: 0, 10, and 50 µM) over a 5-day treatment period. The following parameters were assessed in leaf tissue: photosynthetic performance, antioxidant enzyme activity, levels of nitrogenous compounds, and the expression of genes encoding key enzymes in the arginine-derived metabolic network. Overall, salinity imposed more severe physiological disruptions than copper in both lines, as evidenced by an approximately 88% reduction in photosynthetic performance under 100 mM of salt. Both stresses impaired nitrogen metabolism, increasing ammonia levels and reducing nitrate concentration. Interestingly, *eu3-a* plants demonstrated enhanced tolerance to salt stress relative to *Eu3* plants, but this trend was not observed under copper stress. Future work should address nitrogen-related enzymatic activities associated with urease metabolism and elucidate the non-enzymatic antioxidant mechanisms contributing to stress tolerance in *eu3-a* soybean plants under salt and copper stress.

**Supplementary Information:**

The online version contains supplementary material available at 10.1007/s00425-026-05001-2.

## Introduction

Stress conditions caused by excess copper (Cu) and salt (NaCl) can significantly impact plant physiology, leading to oxidative stress, ion imbalances, and metabolic disruptions (Shabbir et al. 2020; Wang et al. 2024). To counteract these adverse effects, plants activate various anti-stress mechanisms, including the synthesis of polyamines and proline, which play crucial roles in mitigating oxidative damage and maintaining cellular homeostasis (Pál et al. 2018; Ghosh et al. 2022). Polyamines function as reactive oxygen species (ROS) scavengers, while proline contributes to membrane stability and osmotic regulation under stress conditions (Shao et al. 2022).

Copper is an essential micronutrient for plants but also a heavy metal, and when present in excess, it adversely affects plant physiology. In particular, copper excess disrupts nitrogen metabolism by reducing nitrogen uptake through altered expression of genes encoding low-affinity nitrate transporters (NRT1 family) and nitrate reductase (NR1) (Hippler et al. 2018). Cu also leads to a decrease in the enzymatic activities of nitrate reductase (NR) (Xiong et al. 2006; Hussain et al. 2020) and urease (Snieg and Nowak 2005; Han and Wang 2021). In addition, excess copper enhances proline accumulation (Mostofa et al. 2014; Chen et al. 2022; Wang et al. 2024) and increases polyamines levels (Malik et al. 2022).

Polyamines and proline are closely associated with the arginine (Arg) catabolism pathway (Paschalidis et al. 2019). Arg is degraded by the enzyme arginase, producing ornithine (Orn) and urea (Siddappa and Marathe 2020). Urea [(NH_2_)_2_CO], in turn, is hydrolyzed by urease, releasing two molecules of ammonia (NH_3_) and carbon dioxide (Mazzei et al. 2020). The degradation of urea derived from Arg forms ammonia, which is then assimilated by the GS/GOGAT enzymatic system, recycling nitrogen (Witte 2011; Paschalidis et al. 2019).

Both Arg and Orn can be converted into polyamine putrescine (Put) through the action of the enzymes arginine decarboxylase and ornithine decarboxylase, respectively. Putrescine serves as a precursor for other polyamines, such as spermidine and spermine, which are also involved in stress responses (Paschalidis et al. 2019). Proline can be synthesized either from Orn, which is directly involved in Arg catabolism, or from glutamate (Glu), produced, for example, via the GS/GOGAT enzymatic system (Witte 2011; Ghosh et al. 2022).

In soybean, urease plays a central role in nitrogen metabolism by linking different nitrogen metabolic pools (Polacco et al. 2013) and occurs as two isoenzymes: a ubiquitous, constitutive form present in all tissues, and an embryo-specific form synthesized during seed development (Polacco et al. 1982; Polacco and Winkler 1984).

Plant urease is a nickel-dependent metalloenzyme containing two Ni atoms in its active site (Alfano and Cavazza 2020) and requires the accessory proteins UreD, UreF, and UreG for activation, with UreG mediating Ni insertion (Witte 2011; Farrugia et al. 2013). Accordingly, the soybean *eu3-a* mutant is completely urease-negative, as the deletion of *eu3-a* prevents UreG production and, consequently, urease activation (Freyermuth et al. 2000; Polacco et al. 2011; Tezotto et al. 2016).

Studies have shown that the lack of urease activity affects the response to abiotic stresses. Bu et al. (2015), working with a urease mutant in *Arabidopsis thaliana* (Aturease—At1g67550) and a urease inhibitor, found that under saline stress (NaCl), blocking urease activity improves NaCl tolerance during seed germination due to a reduction in NH_4_ concentration. Kawakami et al. (2013) also used a urease inhibitor in cotton plants and found that under saline stress (NaCl), responses differed depending on the NaCl concentration. At low concentrations, urease inhibition makes plants more tolerant, but the same is not observed at high concentrations, due to reduced N assimilation.

Thus, it is evident that Arg degradation and urease activity play fundamental roles in plant responses to environmental stresses. We hypothesize that urease deficiency affects nitrogen metabolism–dependent physiological and antioxidant responses, thereby influencing soybean tolerance to saline and copper stress. Accordingly, this study used the urease-null *eu3-a* mutant to evaluate the effects of salinity and copper stress on urease-related nitrogen metabolism in soybean plants. Understanding this interaction may provide important insights into plant adaptive mechanisms to these stresses and their relationship with nitrogen metabolism.

## Materials and methods

### Plant material and experimental conditions

Seeds from a soybean (*Glycine max* (L.) Kerr) mutant deficient in the urease enzyme (*eu3-a*) and control seeds from the dominant homozygous (*Eu3*) were used in this study. These genotypes were generated using the near-isogenic line (NIL) technique in the Williams/Williams82 genetic background (Tezotto et al. 2016). Two distinct experiments were carried out, one focusing on saline stress and the other on copper stress. The experiments were conducted at the Department of Plant Biology of the UNICAMP in Campinas (São Paulo, Brazil), with the geographic coordinates 22° 49′ 45″ S, 47° 06′ 33″ W and an average altitude of 670 m. In both experiments, the seeds were germinated and cultivated in 1 L pots filled with vermiculite. Plants were maintained under greenhouse conditions throughout the experimental period, during the spring and summer seasons in Brazil, under natural light and ambient temperature conditions, with mean air temperatures ranging between 15 °C (minimum) and 35 °C (maximum), with a medium temperature approximately 24 °C. The photoperiod ranged from approximately 12 h at the beginning of spring to a maximum of about 13 h at the summer solstice. Relative humidity in the greenhouse varied seasonally, being approximately 60% during spring and around 80% during the rainy summer period. All plants were irrigated with 100 mL of Hoagland solution (Hoagland and Arnon 1938), using nitrate as the source of nitrogen, twice a week, or only water as needed.

The plants were evaluated upon reaching their reproductive stage (R2) as this phase is marked by increased nitrogen cycling and heightened activity in the Arg catabolism pathway (Souza et al. 2020a, b). At the end of each experiment, several assessments were conducted: plant biometry, biochemical analyses (oxidative stress and antioxidant metabolism), concentration of nitrate, ammonium, amino acids, polyamines and urea, and the relative expression of genes related to the Arg degradation pathway.

### Treatments: induction of saline and copper stresses

In both experiments, once the plants reached the R2 stage, they were transferred to a hydroponic system and, after a 3-day acclimatization period, they underwent sodium chloride (NaCl) or copper chloride (CuCl_2_) treatments. For the saline stress experiment, the treatments included a control group without NaCl, and 50 mM or 100 mM NaCl. In the copper stress experiment, the treatments involved a control group without copper, and 10 µM or 50 µM CuCl_2_. Throughout both experiments, the nutrient solution pH was continuously monitored and maintained at 5.5, and the solution was renewed every 3 days. The hydroponic system was continuously aerated using an air compressor. Following a 5-day treatment period, leaf samples were collected, promptly immersed in liquid nitrogen, and stored at − 80 °C for biochemical analysis.

### Leaf gas exchange

One day before collection, gas exchange measurements were conducted on the central leaflet of the third fully expanded leaf using an infrared gas analyzer (Li-6400, Licor, Lincoln, NE, USA) in a 6 cm^2^ leaf area. Leaf CO_2_ assimilation (*A*) and stomatal conductance (*g*_s_) were assessed under photosynthetically active radiation of 800 μmol m^−2^ s^−1^ and a partial air CO_2_ pressure of 400 μbar. Measurements were conducted between 9:00 and 12:00 h as reported by Laira et al. (2023).

### Biometry

On the collection day, leaf area measurements were performed using a leaf area meter (LICOR model Li-3100). Then, leaf, stem, and root samples were weighed to determine their fresh weight, and the dry weight was obtained after four days drying in an oven with forced-air circulation at 50 °C.

### Amino acids, polyamines, and urea concentrations

On the collection day, the third and fourth leaves were harvested and ground into a fine powder using liquid nitrogen. A 0.2 g sample of the ground leaf material was used for metabolite extraction with MCW (methanol, chloroform, water, 12:5:3, by vol) according to Bieleski and Turner (1966). The MCW extract was stored at − 20 °C until analysis.

The separation and analysis of amino acids were performed using the *O*-phthalaldehyde (OPA) method (Puiatti and Sodek 1999; Souza et al. 2016). In this method, amino acids are derivatized into their OPA derivatives and subsequently separated by high-performance liquid chromatography (HPLC) on a Spherisorb ODS-2 column (5 μm, 4 × 250 mm). The column was eluted at a flow rate of 0.8 mL min⁻^1^ using a linear gradient of 65% methanol and phosphate buffer at pH 7.25. The column effluent was monitored using a Shimadzu RF-350 fluorescence detector (Shimadzu Corp., Kyoto, Japan) with excitation and emission wavelengths set at 250 nm and 480 nm, respectively. Pure amino acids (AAS18—Sigma) were used to produce standard curves. Glutamine, asparagine, and GABA were added to these pure amino acids standard at 2.5 µmol/mL.

In the same extracts, polyamines, agmatine (Agm) spermine (Spm), spermidine (Spd) and Put, urea and the amino acids Orn, citrulline (Ctr), proline, and tryptophan (Trp) were analyzed by UPLC-MS in a Waters Acquity C18 BEH analytical column (150 mm 9 2.1 mm i.d, 1.7 lm) at 30 °C. Methanol and 0.1% aqueous formic acid were used as mobile phase at a flow rate of 0.2 L min^−1^. Mass spectrometry was carried out using electrospray ionization in the negative mode and the following conditions: capillary 3.0 kV, cone 30 V, ionization source at 150 °C, desolvation temperature of 350 °C, and CID of 15 eV. (Acquity UPLC-MS-Micromass-Waters, Manchester, UK). The data were acquired between 50 and 300 m/z. Ions were identified by the comparison of their m/z, retention time and ESI(+)-MS/MS dissociation patterns with pure standards (Mokochinski et al. 2013; Tezotto et al. 2016).

### Nitrate and ammonium concentrations

Free NH_4_^+^ was determined by the phenol–hypochlorite reaction according to Felker (1977). Nitrate was determined according to Cataldo et al. (1975), with potassium nitrate (KNO_3_) as standard.

### Lipid peroxidation and hydrogen peroxide

Lipid peroxidation was measured as the amount of malondialdehyde (MDA) in the tissues; 0.2 g of fresh leaves were used for extraction in 0.1% trichloroacetic acid and used to determine the thiobarbituric acid reactive substances concentration (Cakmak and Horst 1991). To determine the concentration of MDA, an extinction coefficient of 155 mmol^−1^ cm^−1^ was used.

Hydrogen peroxide (H_2_O_2_) quantification followed the method of Alexieva et al. (2001) using the same extract used for MDA analysis. The reaction medium consisted of 1 mM potassium iodide, 0.1 M potassium phosphate buffer (pH 7.5), and the crude extract. The tubes were kept on ice in the dark for 1 h, after which absorbance was measured at 390 nm. H_2_O_2_ concentrations were determined from a calibration curve using H_2_O_2_ standards and reported as μmol H_2_O_2_ g^−1^.

### Enzyme antioxidant activities

Leaf extracts were prepared by grinding 100 mg of fresh samples with liquid nitrogen, 1% polyvinyl-polypyrrolidone (PVPP), and an extraction medium consisting of 0.1 M potassium phosphate buffer (pH 6.8), 0.1 mM EDTA, and 1 mM phenyl-methane-sulfonyl fluoride (PMSF). After centrifugation at 15,000*g* for 15 min at 4 °C, the supernatants were collected and kept on ice. These extracts were used to determine the activities of superoxide dismutase activity (SOD), catalase activity (CAT), and ascorbate peroxidase activity (APX). The same reaction medium was used for CAT and SOD, which contained 0.1 M potassium phosphate buffer (pH 6.8), 0.1 mM EDTA, 1 mM PMSF, and 1% PVPP. For APX, the specific medium comprised 50 mM potassium phosphate buffer (pH 7.0), 1 mM ascorbic acid, and 1 mM EDTA (Nakano and Asada 1981). SOD activity was determined following the protocol of Giannopolitis and Ries (1977) by measuring NBT photo-reduction inhibition. CAT activity was quantified using the method described by Havir and McHale (1987), and the reaction was conducted at 25 °C for 2 min, with absorbance reduction at 240 nm measured. APX activity was assessed as per Nakano and Asada (1981), with the reaction taking place in a water bath at 25 °C for 2 min. These enzymatic activities were expressed in absorbance units per minute per milligram of fresh weight (FW).

### Gene expression analysis

The relative expression of genes related to the Arg degradation pathway was analyzed. Table S1 describes these genes along with their respective sequences from the soybean genome (*Glycine max* Wm82.a2.v1) available on Phytozome (http://www.phytozome.net). Primers were designed for real-time PCR analysis (qRT-PCR) using the Primer3 Plus^®^ program (http://frodo.wi.mit.edu/primer3/). Only the expressed genes were presented (Table S1). ELF-B and ACT-II or CYP-2 were used as reference genes (Jian et al. 2008). Primer efficiency was evaluated using standard curves generated from serial cDNA dilutions, with amplification efficiencies higher than 94% for all primer pairs. For salt stress, ELF-B and CYP-2 were used as reference genes, whereas for copper stress, ELF-B and ACT-II were used. Amplification specificity was confirmed by melting curve analysis, showing a single peak for all reactions.

RNA extraction was performed using Sigma^®^ TRI Reagent following the manufacturer's instructions. After extraction, the RNA underwent DNase treatment and 1 µg of total RNA was used to synthesize a first strand cDNA with a BIO-RAD^®^ kit, following the manufacturer's instructions. RT-qPCR reactions were prepared using the BIO-RAD^®^ QuantiFastTM SYBR^®^ Green PCR Kit, following the manufacturer's instructions. RT-qPCR was performed using the StepOnePlus Real-Time PCR detection system (Applied Biosystems). Relative expression quantification was determined by comparing transcriptional expression between the target genes and the reference genes using the 2^−ΔCt^ method (Livak and Schmittgen 2001).

### Statistical design and data analysis

The experiments were conducted separately (saline and copper stresses), both following a completely randomized experimental design with five replicates, where each plant was an experimental unit. Data was subjected to analysis of variance, and the means were compared using the Tukey’s test at a 5% probability level.

## Results

### Salt stress

#### Leaf gas exchange and biometry

**Fig. 1:**
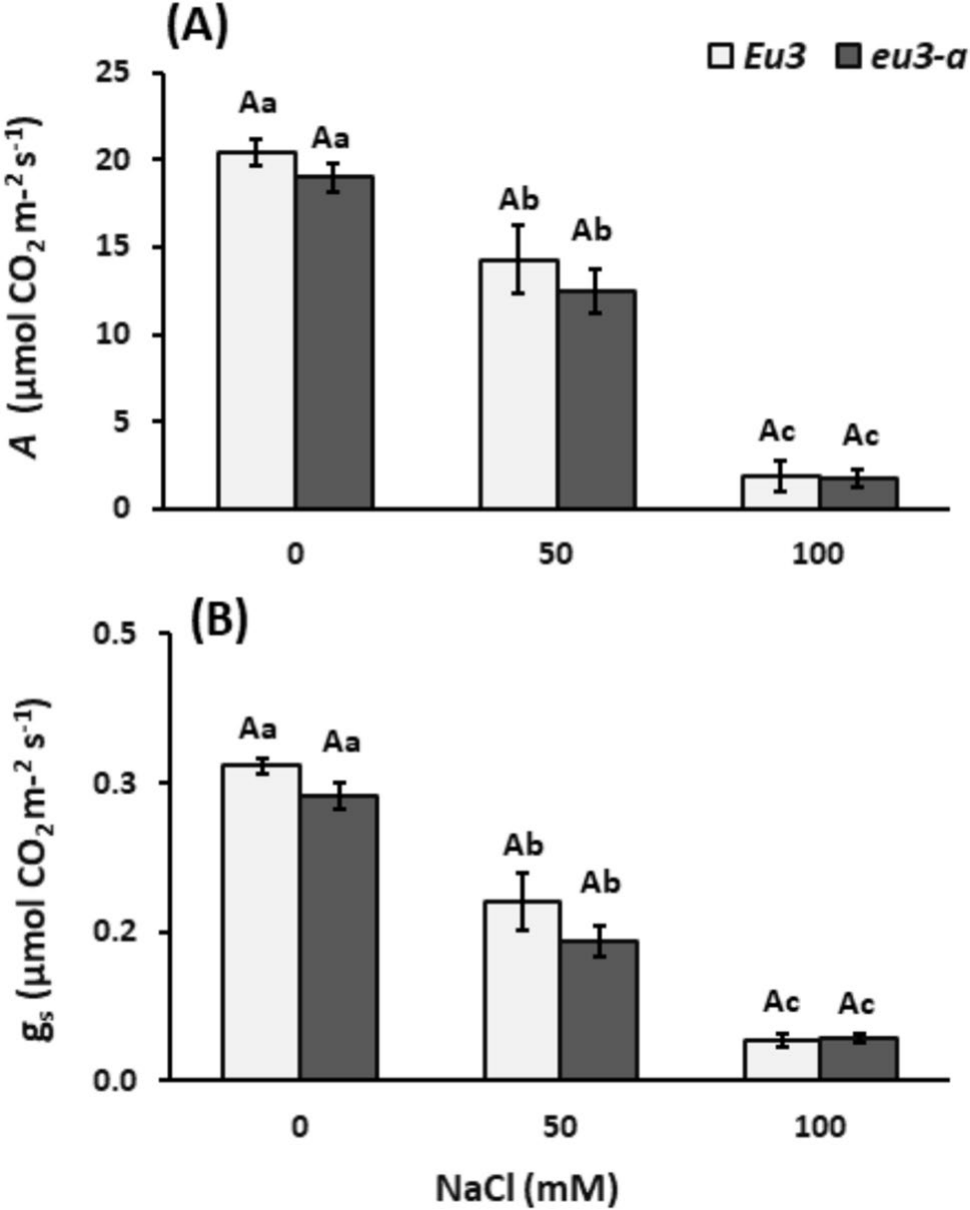
Leaf CO_2_ assimilation (**A**) and stomatal conductance (g_s_, **B**) in *Eu3* and *eu3-a* plants under different NaCl concentrations (0, 50, and 100 mM). The values represent the mean of five replications ± standard error. Uppercase letters denote comparisons between NILs within each treatment, while lowercase letters indicate comparisons between treatments within each NIL (Tukey’s test, *P* < 0.05)

Both NILs exhibited stomatal conductance and photosynthesis significantly influenced by NaCl treatments in a dose-dependent manner. Nevertheless, no differences were noted between the NILs (Fig. 1). Regarding biometric data, leaf area, leaf fresh mass, and root mass were reduced under the 100 mM NaCl treatment in *Eu3* plants (control). Nevertheless, no differences were observed between the NILs (Table 1). Notably, only the fresh mass was affected, highlighting that the water content—indicative of cell turgidity in these organs—was impacted across the *Eu3* plant treatments.

**Table 1:** Biometric measurements of *Eu3* and *eu3-a* plants (NILs) with increasing NaCl concentrations (0, 50, and 100 mM). Measurements were made at the end of the experiment (5 days)

NILs	NaCl (mM)	LA (cm^2^)	LFM (g)	RFM (g)	SFM (g)	LDM (g)	RDM (g)	SDM (g)
*Eu3*	0	297.1 ± 31.7 Aab	5.4 ± 0.5 Aa	7.0 ± 0.8 Aab	4.5 ± 0.6 Aa	1.1 ± 0.07 Aa	0.6 ± 0.06 Aa	0.8 ± 0.12 Aa
	50	318.2 ± 10.5 Aa	5.6 ± 0.2 Aa	6.8 ± 0.3 Aa	4.3 ± 0.2 Aa	1.1 ± 0.23 Aa	0.5 ± 0.12 Aa	0.7 ± 0.17 Aa
	100	199.6 ± 30.8 Ab	2.6 ± 0.7 Ab	3.8 ± 1.0 Ab	3.7 ± 0.9 Aa	1.1 ± 0.29 Aa	0.6 ± 0.15 Aa	0.8 ± 0.18 Aa
*eu3-a*	0	292.7 ± 28.5 Aa	5.4 ± 0.6 Aa	6.5 ± 0.6 Aa	4.5 ± 0.2 Aa	1.1 ± 0,03 Aa	0.6 ± 0.03 Aa	0.7 ± 0.07 Aa
	50	355.8 ± 86.1 Aa	6.1 ± 1.5 Aa	7.1 ± 1.6 Aa	4.5 ± 1.0 Aa	1.1 ± 0.09 Aa	0.5 ± 0.03 Aa	0.7 ± 0.03 Aa
	100	210.6 ± 51.0 Aa	4.6 ± 0.9 Aa	4.9 ± 0.6 Aa	3.6 ± 0.3 Aa	1.0 ± 0.17 Aa	0.4 ± 0.03 Aa	0.7 ± 0.06 Aa

Data represent mean values (*n* = 5) ± standard error. Uppercase letters represent the comparison between NILs within each treatment and lowercase letters represent the comparison between treatments within each NILs (Tukey test, *P* < 0.05)

LA, leaf area; LFM, leaf fresh mass; RFM, root fresh mass; SFM, stem fresh mass; LDM, leaf dry mass; RDM, root dry mass; SDM, stem dry mass

#### Oxidative damage and antioxidant metabolism

**Fig. 2:**
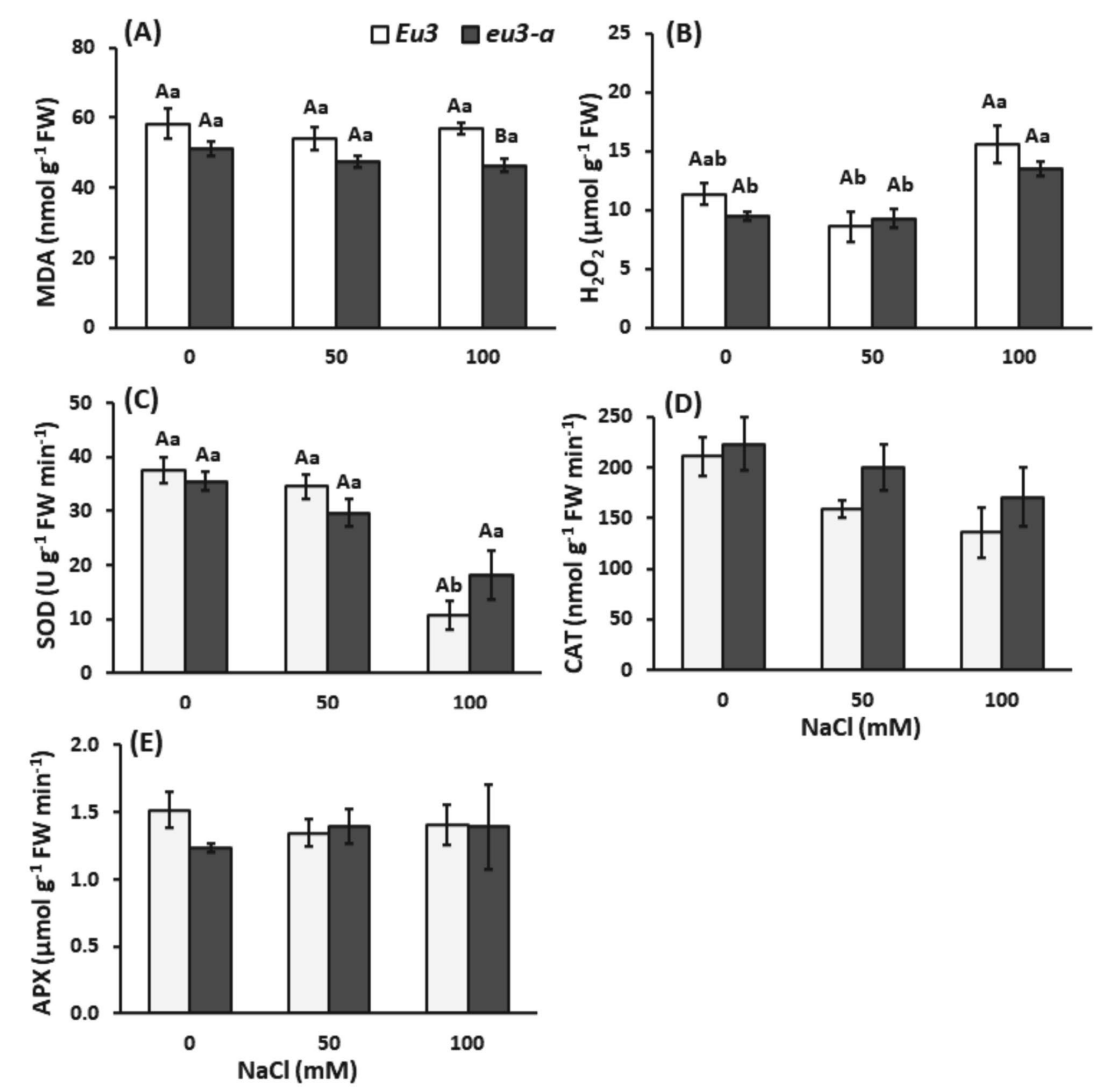
Concentration of malondialdehyde (MDA, **A**), hydrogen peroxide (H_2_O_2_, **B**), activities of superoxide dismutase (SOD, **C**), catalase (CAT, **D**) and ascorbate peroxidase (APX, **E**) in leaves of *Eu3* and *eu3-a* plants under different NaCl concentrations (0, 50, and 100 mM). The values represent the mean of five replications ± standard error. Uppercase letters denote comparisons between NILs within each treatment, while lowercase letters indicate comparisons between treatments within each NIL (Tukey test, *P* < 0.05)

The concentration of hydrogen peroxide (H_2_O_2_) was affected by the NaCl treatment, showing an increase in the 100 mM NaCl treatment for both NILs, nevertheless, without differences between NILs (Fig. 2B). Concerning MDA, no differences were observed among NaCl treatments. However, at the 100 mM treatment, *Eu3* plants exhibited higher MDA levels than *eu3-a* plants. Thus, *Eu3* plants appear to be more susceptible to lipid peroxidation than *eu3-a* plants (Fig. 2A).

In general, SOD activity decreased in the leaves of *Eu3* plants under the 100 mM NaCl treatment (Fig. 2C). CAT and APX enzymes remained unaffected by the saline treatment and showed no differences between the NILs (Fig. 2D, E). Although there seems to be a tendency for a reduction in CAT activity, indicated by decreasing means with increasing NaCl and lower values in *Eu3* plants, this difference was not statistically significant (Fig. 2D).

#### Nitrogen compounds

Concerning amino acids, no differences were observed between NILs, but variations occurred among NaCl treatments (Table 2). Generally, there was an elevation in free amino acid concentration, indicating an overall increase in total amino acids for both NILs. Exceptions were observed for Asp, Ctr, and Orn, which decreased in the 100 mM treatment, while Ala and GABA (gamma-aminobutyric acid) remained unaffected by treatments in both NILs. Notably, Trp, Ser, and proline significantly increased in the 100 mM treatment (10, 5, and 3 times, respectively, compared to the control). The total free amino acid concentration nearly doubled in the 100 mM treatment compared to the control (Table 2).

**Table 2:** Free amino acids content (nmol g^−1^ FW) and total free amino acids (µmol g^−1^ FW) of *Eu3* and *eu3-a* plants (NILs) with increasing NaCl concentrations (0, 50, and 100 mM). Measurements were made at the end of the experiment (5 days)

Amino acids								
NILs	NaCl (mM)	Glu	Asp	GABA	Asn	Ser	Ala	Others
*Eu3*	0	1210.9 ± 108.5 Aab	570.0 ± 84.3 Aa	434.9 ± 88.0 Aa	334.1 ± 40.5 Ab	247.4 ± 22.6 Ab	240.7 ± 29.7 Aa	249.4 ± 56.8 Ab
	50	740.6 ± 49.1 Aa	153.0 ± 11.9 Ab	536.0 ± 96.2 Aa	360.1 ± 57.8 Aab	698.8 ± 69.0 Aab	204.5 ± 29.9 Aa	325.2 ± 47.2 Aab
	100	1686.9 ± 145.8 Ab	222.0 ± 18.5 Ab	514.1 ± 67.4 Aa	618.8 ± 73.5 Aa	1495.0 ± 115.0 Aa	201.8 ± 6.9 Aa	1658.7 ± 280.3 Aa
*eu3-a*	0	771.3 ± 20.0 Ab	359.6 ± 12.9 Aa	608.2 ± 42.8 Aa	289.2 ± 32.8 Aa	258.4 ± 11.2 Ab	250.3 ± 11.3 Aa	231.5 ± 28.8 Ab
	50	878.0 ± 79.9 Aa	179.0 ± 11.4 Aa	578.4 ± 59.4 Aa	431.7 ± 24.3 Aa	778.6 ± 36.1 Aab	214.5 ± 27.8 Aa	350.6 ± 41.9 Ab
	100	1761.7 ± 272.0 Aa	231.3 ± 43.4 Aa	429.9 ± 47.0 Aa	758.9 ± 199.5 Aa	1387.0 ± 239.8 Aa	194.5 ± 24.4 Aa	1933.9 ± 470.9 Aa

NILs	NaCl (mM)	Gln	Trp	Orn	Pro	Ctr	Arg	TOTAL
*Eu3*	0	198.7 ± 27.3 Aa	106.6 ± 7.4 Ab	106.4 ± 14.0 Aab	40.4 ± 2.8 Ab	31.9 ± 1.8 Aa	29.9 ± 4.8 Ab	3.5 ± 0.4 Aab
	50	238.3 ± 28.3 Aa	166.6 ± 21.4 Ab	138.5 ± 18.5 Aa	32.2 ± 0.7 Ab	25.6 ± 0.8 Aab	25.7 ± 3.3 Ab	3.3 ± 0.3 Ab
	100	309.9 ± 29.5 Aa	1097.0 ± 163.2 Aa	66.8 ± 6.8 Ab	140.2 ± 10.2 Aa	17.8 ± 0.6 Ab	65.9 ± 10.1 Aa	6.7 ± 0.6 Aa
*eu3-a*	0	153.2 ± 15.0 Ab	107.3 ± 12.5 Ab	117.9 ± 14.2 Aab	38.2 ± 0.9 Ab	30.5 ± 0.9 Aa	23.5 ± 2.9 Ab	3.0 ± 0.1 Ab
	50	225.7 ± 25.6 Aab	141.7 ± 9.6 Ab	135.7 ± 6.2 Aa	35.2 ± 0.6 Ab	23.5 ± 0.8 Aab	23.9 ± 0.7 Ab	3.7 ± 0.1 Ab
	100	297.7 ± 39.0 Aa	1362.4 ± 341.3 Aa	62.0 ± 10.2 Ab	176.2 ± 19.8 Aa	18.3 ± 1.1 Ab	75.5 ± 17.8 Aa	7.0 ± 1.3 Aa

Data represent mean values (*n* = 5) ± standard error. Uppercase letters represent the comparison between NILs within each treatment and lowercase letters represent the comparison between treatments within each NILs (Tukey test, *P* < 0.05)

Glu, glutamate; Asp, aspartate; GABA, gamma-aminobutyric acid; Asn, asparagine; Ser, serine; Ala, alanine; Gln, glutamine; Trp, tryptophan; Orn, ornithine; Pro, proline; Ctr, citrulline; Arg, arginine; others includes glycine, threonine, histidine, tyrosine, methionine, valine, and lysine

**Fig. 3:**
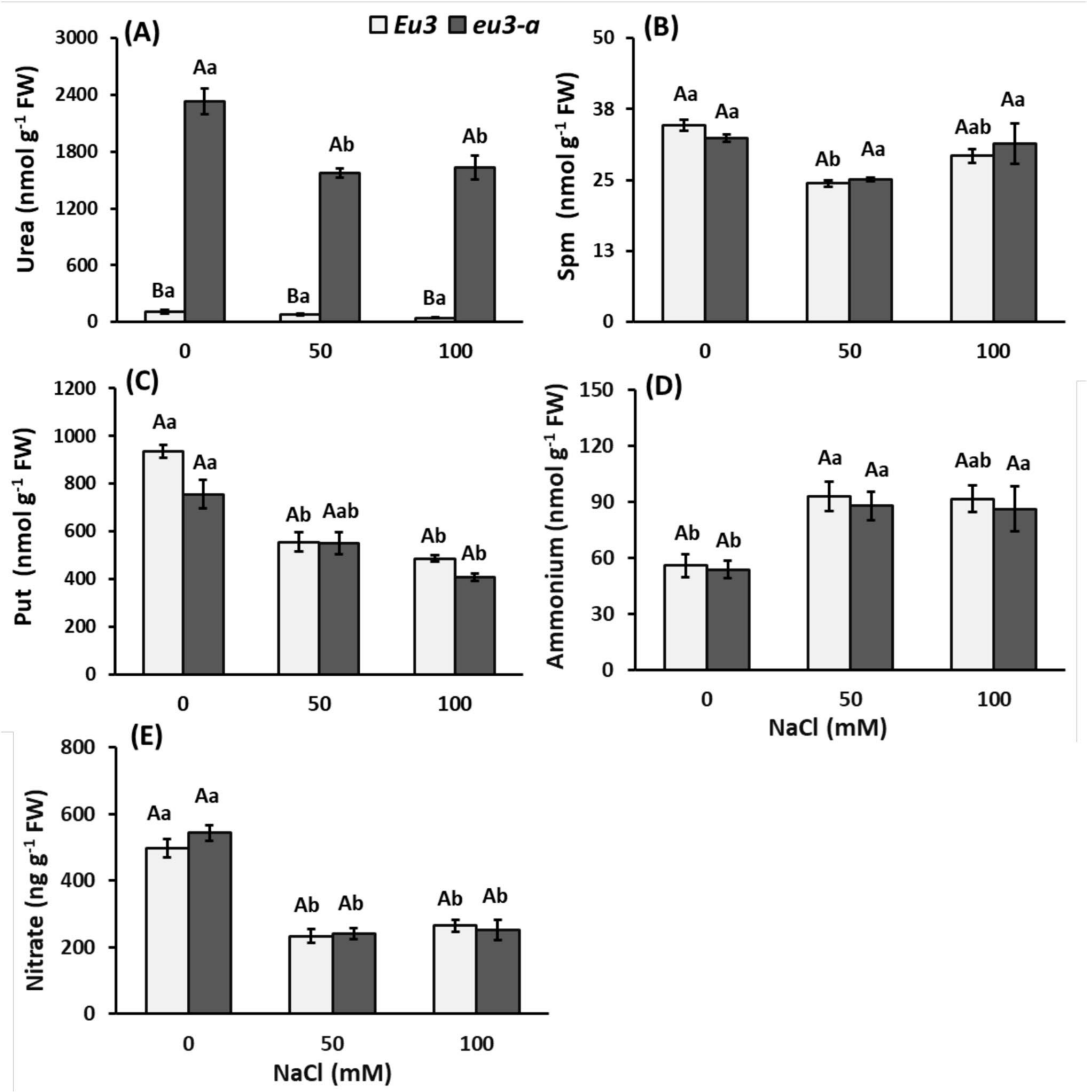
Urea (**A**), polyamines spermine (Spm, **B**) and putrescine (Put, **C**), ammonium (**D**) and nitrate (**E**) contents in leaves of *Eu3* and *eu3-a* plants under different NaCl concentrations (0, 50, and 100 mM). The values represent the mean of five replications ± standard error. Uppercase letters denote comparisons between NILs within each treatment, while lowercase letters indicate comparisons between treatments within each NIL (Tukey test, *P* < 0.05)

The urea concentration in the leaves of *eu3-a* plants was more than 20 times higher than in *Eu3* plants (Fig. 3A). Considering the NaCl treatments, no difference was observed among *Eu3* plants, whereas in *eu3-a* plants, urea concentration was higher in the control than 50 mM and 100 mM treatments (Fig. 3A). In *Eu3* plants, there was a reduction in Spm in the 50 mM treatment, but an increase in the 100 mM treatment (Fig. 3B). It was observed a decrease in Put in both NILs (Fig. 3C) while Agm and Spd were not detected. Ammonium and nitrate concentrations were also quantified. Ammonium levels were elevated in the 50 mM and 100 mM treatments for both NILs (Fig. 3D). Conversely, nitrate concentration showed a reduction in these treatments (Fig. 3E). Notably, the ammonium concentration exhibited no differences between the NILs despite the *eu3-a* mutant's inability to metabolize urea.

#### Genes associated with the Arg metabolism degradation pathway

**Fig. 4:**
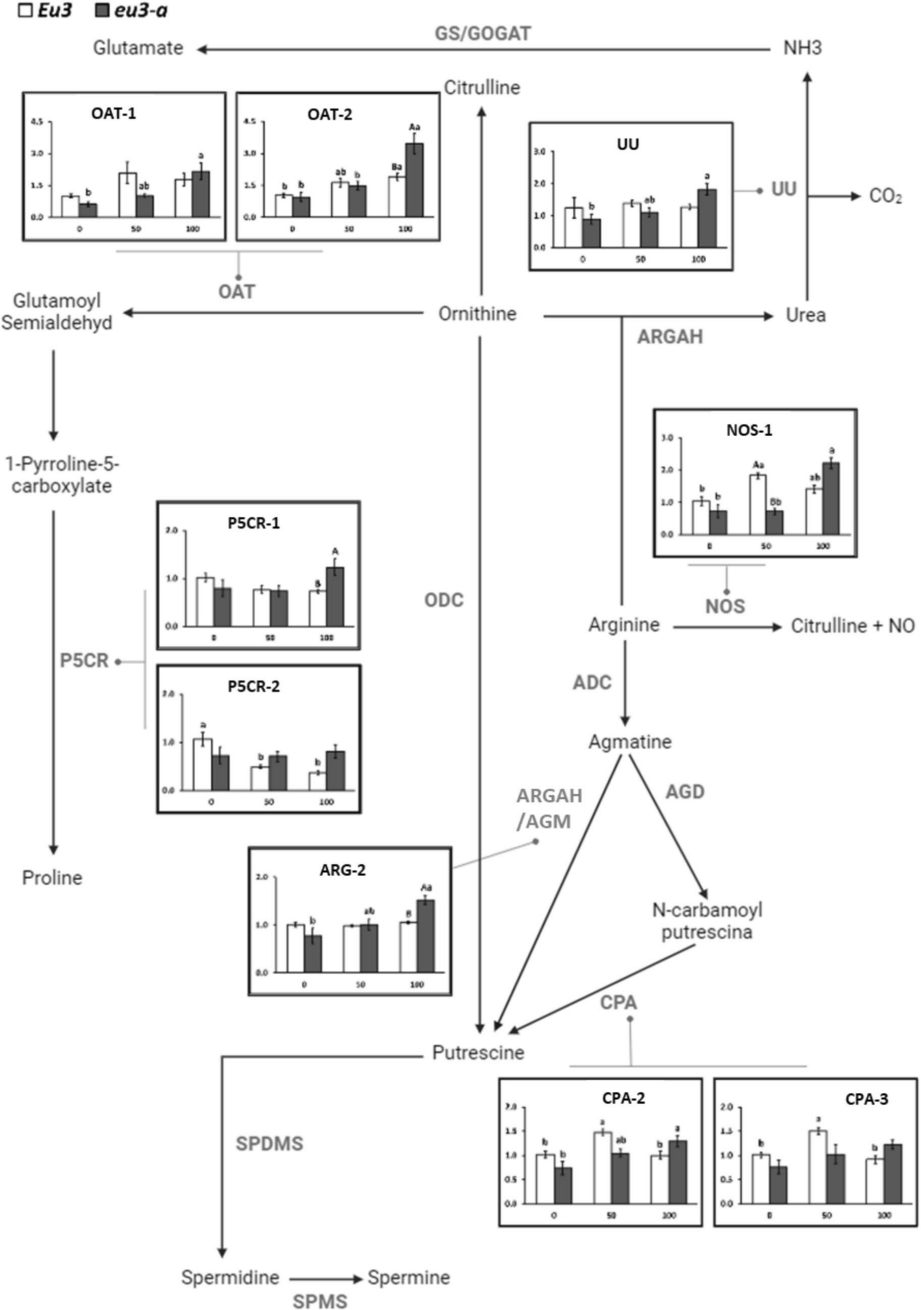
Overview of genes associated with the arginine metabolism degradation pathway. Relative expression (ΔΔCT) of these genes in leaves of *Eu3* and *eu3-a* plants under different NaCl concentrations (0, 50, and 100 mM). The values represent the mean of five replications ± standard error. Uppercase letters denote comparisons between NILs within each treatment, while lowercase letters indicate comparisons between treatments within each NIL (Tukey test, *P* < 0.05). OAT, ornithine aminotransferase; UU, ubiquitous urease; ARGAH, arginase; P5CR, pyrroline-5-carboxylate reductase; NOS, nitric oxide synthase like; CPA, N-carbamoylputrescine amidohydrolase; AGD, agmatine deiminase; ADC, Arginine decarboxylase

Notable changes were observed in the expression of genes related to Arg catabolism pathway, particularly under 100 mM NaCl dose, with a pronounced effect in *eu3-a* plants (Fig. 4). In *eu3-a* plants, the genes associated with ubiquitous urease (UU), arginase (ARGAH-2), ornithine aminotransferase (OAT-1 and OAT-2), *N*-carbamoylputrescine amidohydrolase (CPA-2), and nitric oxide synthase (NOS-1-like) exhibited higher expression levels in the 100 mM treatment. Conversely, in *Eu3* plants, the *OAT-1* gene showed increased expression in the 100 mM treatment, while CPA-2, CPA-3, and NOS-1-like genes exhibited higher expression in the 50 mM treatment.

## Copper stress

### Leaf gas exchange and biometry

**Fig. 5:**
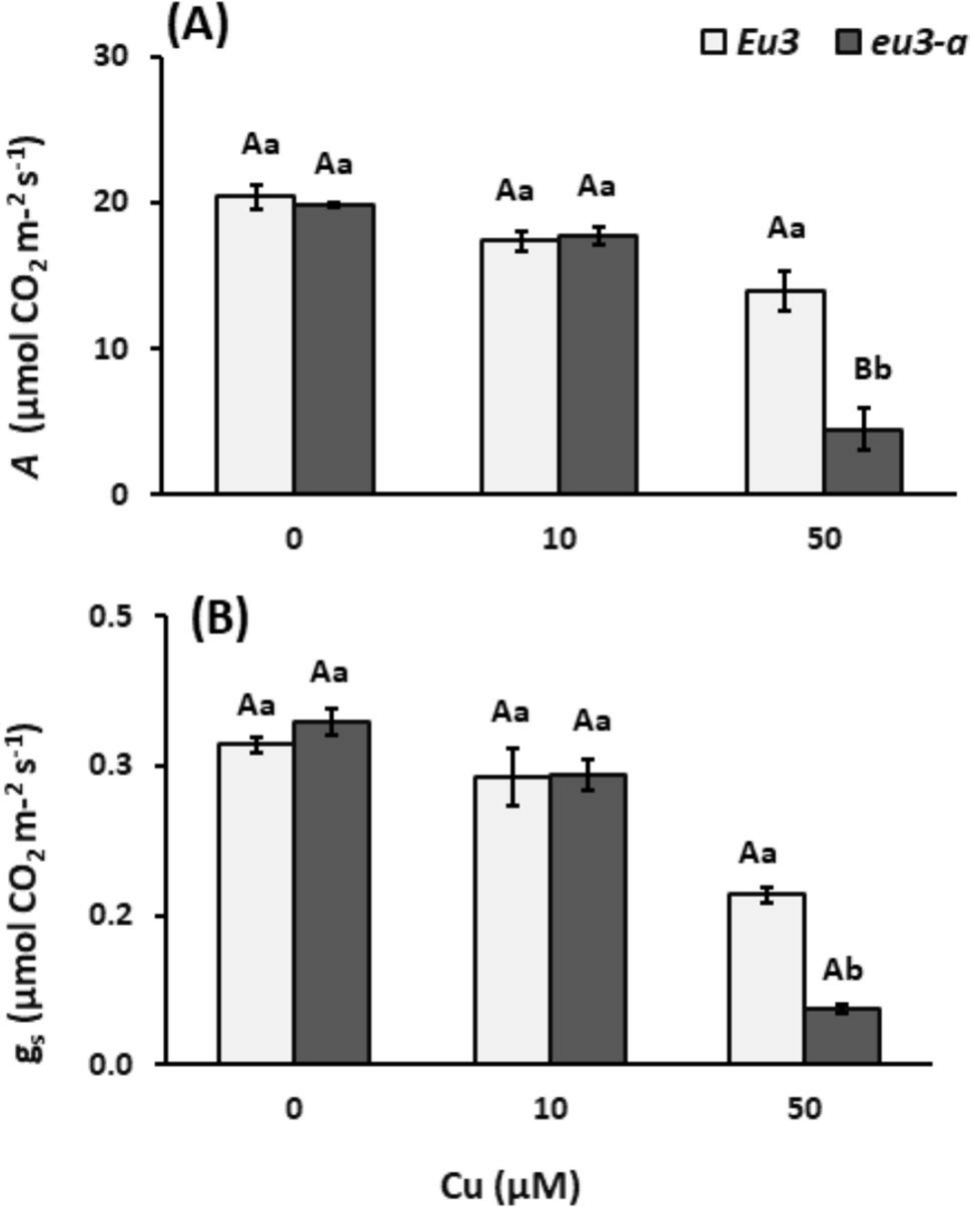
Leaf CO_2_ assimilation (**A**) and stomatal conductance (g_s_, **B**) in *Eu3* and *eu3-a* plants under different copper concentrations (0, 10, and 50 µM). The values represent the mean of five replications ± standard error. Uppercase letters denote comparisons between NILs within each treatment, while lowercase letters indicate comparisons between treatments within each NIL (Tukey test, *P* < 0.05)

Unlike plants subjected to saline stress, photosynthesis and stomatal conductance exhibited reductions only in *eu3-a* plants exposed to the 50 µM Cu treatment (Fig. 5). Furthermore, the biometric data assessed remained unaffected by both copper treatments and NILs (Table S2).

#### Oxidative damage and antioxidant metabolism

**Fig. 6:**
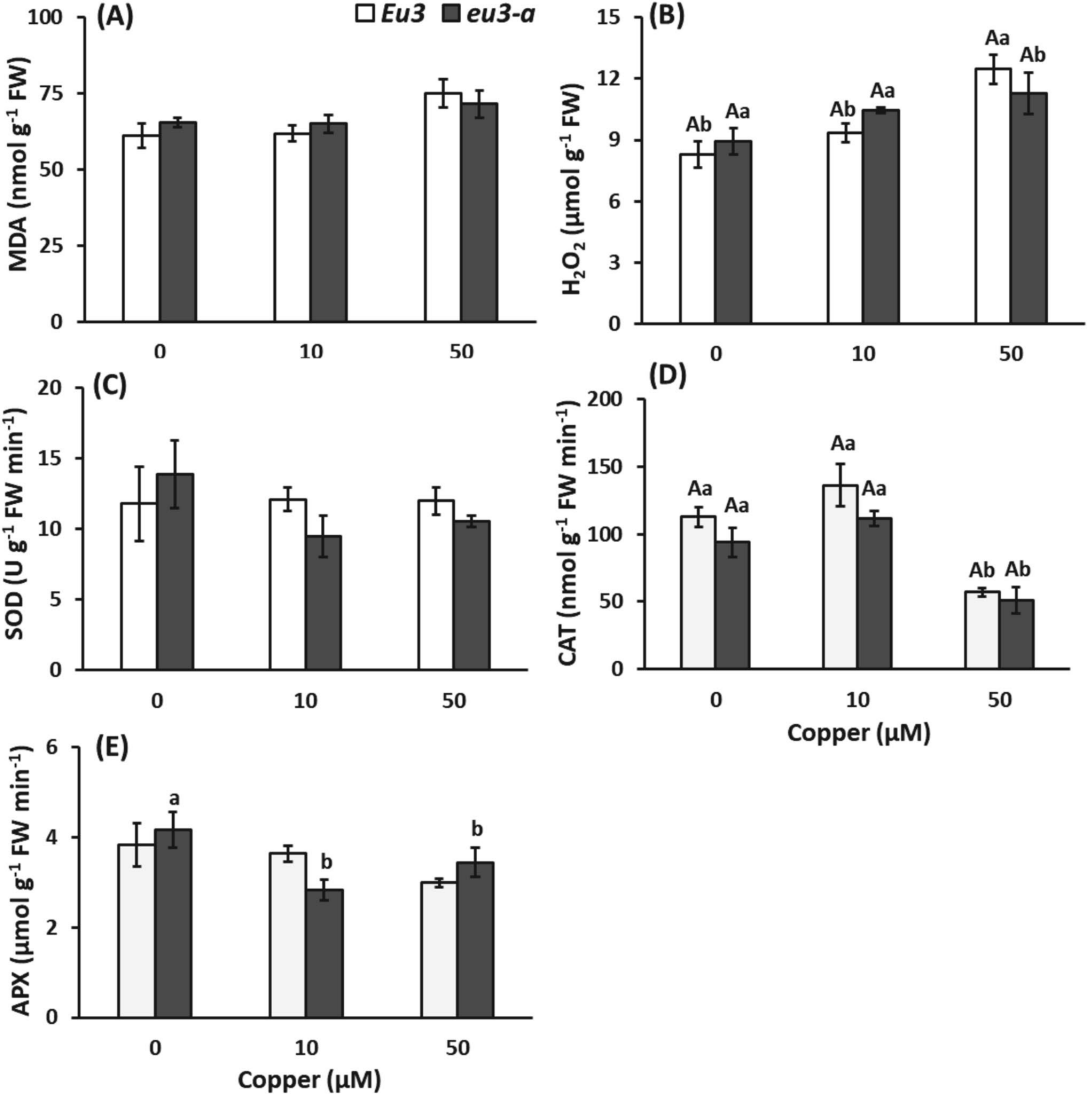
Concentration of malondialdehyde (MDA, **A**), hydrogen peroxide (H_2_O_2_, **B**), activities of superoxide dismutase (SOD, **C**), catalase (CAT, **D**) and ascorbate peroxidase (APX, **E**) in leaves of *Eu3* and *eu3-a* plants under different copper concentrations (0, 10, and 50 µM). The values represent the mean of five replications ± standard error. Uppercase letters denote comparisons between NILs within each treatment, while lowercase letters indicate comparisons between treatments within each NIL (Tukey test, *P* < 0.05)

The MDA concentration remained unaffected by varying copper doses across all NILs (Fig. 6). However, there was an increase in H_2_O_2_ concentration in the leaves of *Eu3* plants under the 50 µM copper treatment (Fig. 6A, B). Regarding antioxidant enzymes, while no significant changes were noted in APX and SOD activities, a decline in CAT activity was observed in response to the 50 µM copper treatment in both NILs (Fig. 6C–E).

#### Nitrogen compounds

**Fig. 7:**
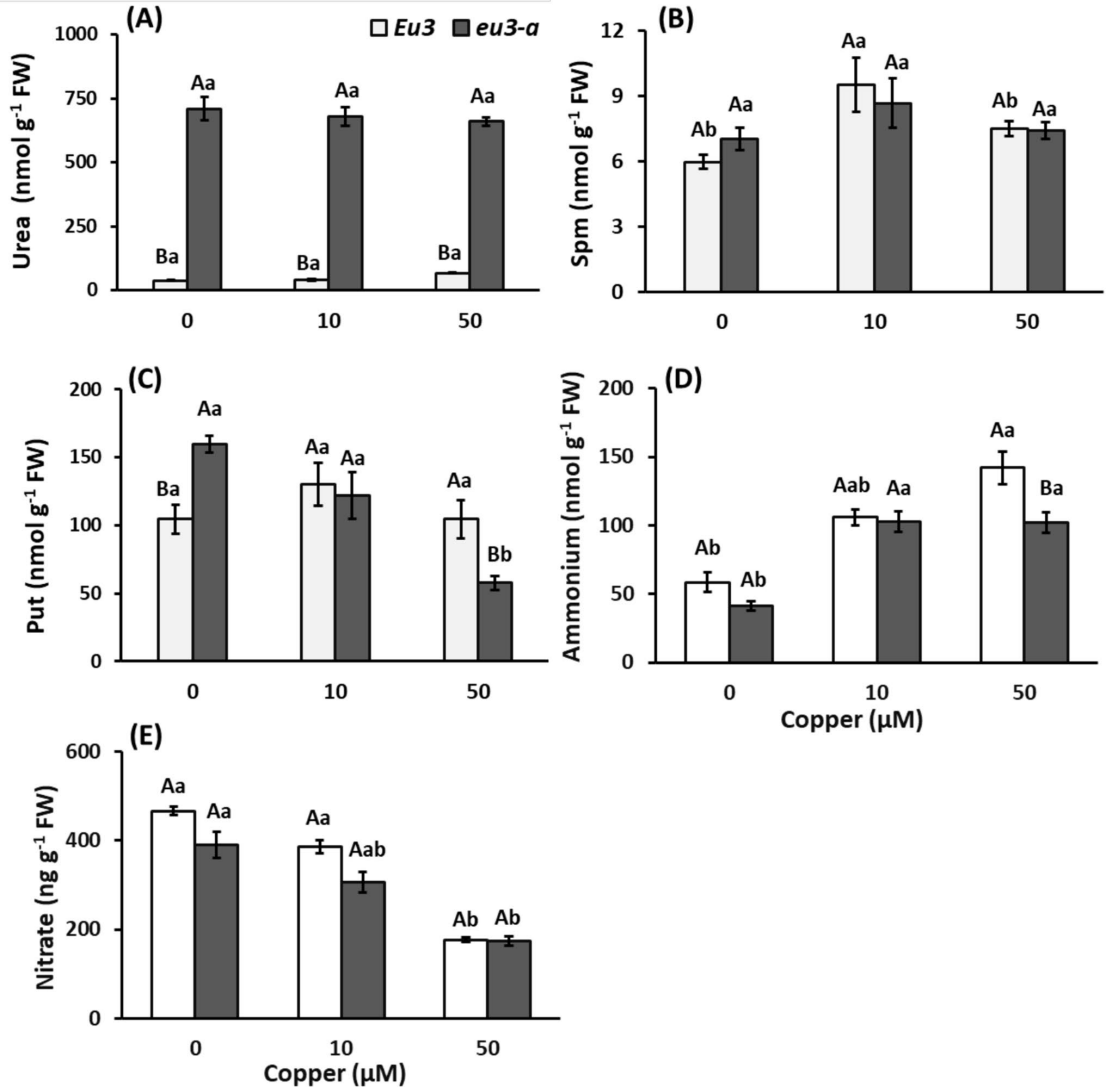
Urea (**A**), polyamines spermine (Spm, **B**) and putrescine (Put, **C**), ammonium (**D**) and nitrate (**E**) contents in leaves of *Eu3* and *eu3-a* plants under different copper concentrations (0, 10, and 50 µM). The values represent the mean of five replications ± standard error. Uppercase letters denote comparisons between NILs within each treatment, while lowercase letters indicate comparisons between treatments within each NIL (Tukey test, *P* < 0.05)

Regarding the amino acid profile, notable changes were detected, particularly in *eu3-a*. This included a reduction in aspartate (Asp), Ala, asparagine (Asn), and Ctr with an escalating copper dose, a trend not observed in *Eu3* plants. Additionally, both NILs exhibited an elevation in Trp at a concentration of 50 µM of copper (Table 3). Concerning urea levels, the leaves of *eu3-a* plants exhibited a significantly higher urea concentration compared to *Eu3* plants, regardless of copper dose (Fig. 7A).

**Table 3:** Free amino acids content (nmol g^−1^ FW) and total free amino acids (µmol g^−1^ FW) of *Eu3* and *eu3-a* plants (NILs) with increasing Cu concentrations (0, 10, and 50 µM). Measurements were made at the end of the experiment (5 days)

Amino acids								
NILs	Cu (µM)	Glu	Asp	GABA	Asn	Ser	Ala	Others
*Eu3*	0	325.3 ± 9.5 Aa	298.7 ± 9.9 Aa	491.8 ± 50.6 Aa	259.0 ± 9.9 Aa	321.2 ± 4.3 Aa	311.1 ± 8.7 Aa	244.1 ± 33.3 Aa
	10	316.2 ± 3.7 Aab	267.3 ± 6.3 Aa	553.2 ± 15.6 Aa	276.2 ± 6.3 Aa	283.4 ± 7.2 Ab	318.7 ± 7.5 Aa	279.2 ± 31.0 Aa
	50	288.5 ± 10.7 Ab	258.5 ± 10.0 Aa	550.3 ± 51.8 Aa	221.6 ± 10.0 Aa	206.7 ± 16.1 Ab	298.1 ± 40.7 Aa	219.1 ± 20.7 Aa
*eu3-a*	0	327.8 ± 4.3 Aa	325.6 ± 10.1 Aa	460.1 ± 37.7 Aa	283.1 ± 10.1 Aa	255.6 ± 20.8 Aa	318.3 ± 7.7 Aa	274.7 ± 28.3 Aa
	10	304.0 ± 12.2 Ab	254.9 ± 24.0 Ab	541.2 ± 24.0 Aa	241.6 ± 24.0 Ab	254.0 ± 42.9 Aa	295.8 ± 17.2 Aab	279.1 ± 51.9 Aa
	50	269.0 ± 5.1 Ab	235.9 ± 22.9 Ab	419.0 ± 39.2 Aa	164.7 ± 22.9 Ab	226.0 ± 12.7 Aa	228.6 ± 24.2 Ab	195.8 ± 21.0 Aa

NILs	Cu (µM)	Gln	Trp	Orn	Pro	Ctr	Arg	TOTAL
*Eu3*	0	189.8 ± 17.3 Aa	13.4 ± 2.4 Ab	0.8 ± 0.06 Aa	33.9 ± 0.8 Ba	7.5 ± 0.7 Aa	29.0 ± 1.6 Ab	2.6 ± 0.05 Aa
	10	190.9 ± 13.3 Aa	12.4 ± 2.0 Ab	0.6 ± 0.05 Aab	34.9 ± 0.8 Aa	8.6 ± 1.6 Aa	63.2 ± 12.9 Aa	2.6 ± 0.06 Aa
	50	168.9 ± 11.9 Aa	43.1 ± 10.9 Aa	0.5 ± 0.04 Ab	31.7 ± 0.8 Aa	5.3 ± 0.9 Aa	37.1 ± 4.5 Ab	2.4 ± 0.08 Aa
*eu3-a*	0	148.0 ± 14.5 Ab	15.1 ± 3.2 Ab	0.8 ± 0.04 Aa	40.6 ± 0.9 Aa	5.0 ± 0.7 Ab	30.7 ± 5.5 Aa	2.5 ± 0.05 Aa
	10	207.2 ± 14.6 Aab	13.7 ± 3.4 Ab	0.4 ± 0.01 Ab	32.5 ± 0.8 Aab	9.9 ± 2.1 Aa	45.4 ± 8.8 Aa	2.6 ± 0.15 Aa
	50	172.2 ± 17.3 Aa	48.9 ± 9.6 Aa	0.4 ± 0.03 Ab	31.4 ± 2.5 Ab	4.3 ± 0.5 Ab	29.4 ± 1.9 Aa	2.1 ± 0.07 Aa

Data represent mean values (*n* = 5) ± standard error. Uppercase letters represent the comparison between NILs within each treatment and lowercase letters represent the comparison between treatments within each NILs (Tukey test, *P* < 0.05)

Glu, glutamate; Asp, aspartate; GABA, gamma-aminobutyric acid; Asn, asparagine; Ser, serine; Ala, alanine; Gln, glutamine; Trp, tryptophan; Orn, ornithine; Pro, proline; Ctr, citrulline; Arg, arginine; others includes glycine, threonine, histidine, tyrosine, methionine, valine, and lysine

There were more Put in the control treatment of *eu3-a* compared to *Eu3* (Fig. 7C). However, at 50 µM of copper, the opposite occurred. Additionally, a reduction of Put in *eu3-a* was observed at 50 µM of copper. Spm did not differ between the NILs, but an increase in concentration was observed in *eu3-a* at 10 µM (Fig. 7B, C). In *Eu3* NILs the ammonium level was elevated in the highest Cu concentration (50 µM). Conversely, nitrate concentration displayed a reduction with increasing copper doses, without distinctions between NILs (Fig. 7D, E).

#### Genes associated with the Arg metabolism degradation pathway

**Fig. 8:**
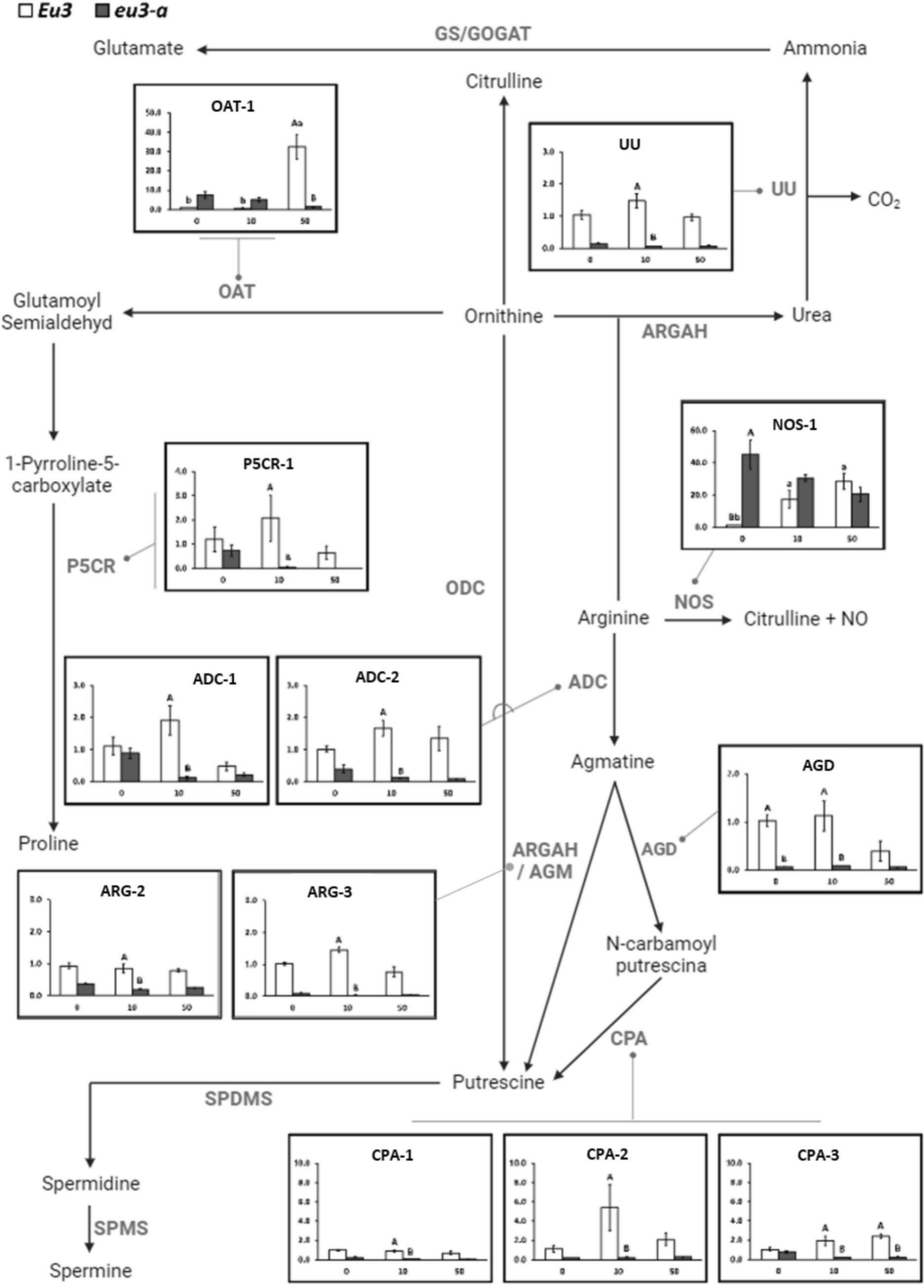
Overview of genes associated with the arginine metabolism degradation pathway. Relative expression (ΔΔCT) of these genes in leaves of *Eu3* and *eu3-a* plants under different copper concentrations (0, 10, and 50 µM). The values represent the mean of five replications ± standard error. Uppercase letters denote comparisons between NILs within each treatment, while lowercase letters indicate comparisons between treatments within each NIL (Tukey test, *P* < 0.05). OAT, ornithine aminotransferase; UU, ubiquitous urease; ARGAH, arginase; P5CR, pyrroline-5-carboxylate reductase; NOS, nitric oxide synthase like; CPA, *N*-carbamoylputrescine amidohydrolase; AGD, agmatine deiminase; ADC, arginine decarboxylase

For most of the evaluated genes, higher expression levels were observed in the *Eu3* genotype compared to the *eu3-a* mutant at 10 µM Cu (Fig. 8). In the *OAT-1* gene, distinctions in copper concentrations were evident in the *eu3-a* genotype, where a heightened expression of *OAT-1* was specifically noted in the 50 µM treatment.

## Discussion

### Salinity caused greater oxidative damage and fresh mass reduction in *Eu3* plants, while both NILs exhibited reduced photosynthesis

Both NILs exhibited a decrease in stomatal conductance and photosynthesis with the increase in NaCl concentration. NaCl stress primarily induces stomatal closure due to diminished cell turgor pressure, thereby limiting CO_2_ availability in leaves, and can also lead to a decline in the photosynthetic electron transport chain (Chaves et al. 2009). The reduction in photosynthesis may be associated with an initial restriction in electron transfer on the acceptor side of PSII, followed by photodamage affecting both PSII and PSI, which together could disrupt overall electron transport (Singh et al. 2016; Huang et al 2019). Additionally, free ammonium levels increased under salt stress. In leaves, photorespiration represents a major source of ammonium, as the mitochondrial conversion of glycine-to-serine releases ammonium (Miao et al. 2024). Accordingly, the increase in Ser content observed under 100 mM NaCl may reflect changes in photo-respiratory flux since Ser is a direct product of this pathway (Rosa-Téllez et al. 2024).

Moderate salt stress is often associated with increased photorespiration, which has been proposed as an adaptive mechanism to dissipate excess energy, prevent over-reduction of the photosynthetic electron transport chain, and limit photo-inhibition (Huang et al. 2019). Enhanced photorespiration may therefore contribute to increased ammonium release, which, if not efficiently re-assimilated, can result in ammonium accumulation (Zhou et al. 2015). Excess ammonium is known to impair plant metabolism through multiple mechanisms, including alterations in chloroplast ultrastructure, disruption of hormonal homeostasis, inhibition of photosynthesis, imbalances in mineral cation uptake, limitations in carbon supply, enhanced proton efflux and pH disturbances, as well as changes in polyamine metabolism and cell wall rigidity (Zhou et al. 2015; Xie et al. 2025).

Salt-induced reductions in photosynthesis may limit reducing power availability, potentially constraining nitrate reduction and ammonium assimilation and contributing to the decreased nitrate levels observed under NaCl (Huang et al. 2019). These responses are consistent with alterations in nitrogen balance and metabolism, frequently reported alongside reductions in NR, GS, and GOGAT activities under saline conditions (Singh et al. 2016; Farhangi-Abriz and Torabian 2018). Beyond serving as a nitrogen source, nitrate also acts as a signaling molecule regulating nitrogen status and the expression of transporters and assimilation enzymes, including GOGAT (Chen et al. 2022).

The significant reduction in the *Eu3* plant fresh matter under the 100 mM NaCl treatment suggests an alteration in water content, indicating compromised cell turgidity. One of the main effects of increasing NaCl in the soil is the reduction of water potential and, consequently, a reduction in water absorption by plants (Chaves et al. 2009).

Thus, salt stress reduces photosynthesis and the supply of reducing power, impairing nitrate reduction and ammonium assimilation. At the same time, photorespiration is enhanced, leading to the release of ammonium and increased serine production. These combined effects favor ammonium accumulation, resulting in metabolic and physiological stress. The consequences include growth inhibition, disturbances in osmotic balance, and shifts in amino acid composition.

Both NILs exhibited higher concentration of H_2_O_2_ at the 100 mM NaCl treatment. This ROS is a moderately reactive oxidant that freely diffuses across membranes and, at high NaCl concentrations, can severely affect cells, inactivate enzymes through oxidation, and cause damage to lipids, nucleic acids, and proteins (Miller et al. 2010).

SOD has been considered the first-line defense against ROS increase due to several abiotic stresses, catalyzing the dismutation of superoxide ions into H_2_O_2_ (Sharma et al. 2012). SOD was reduced in the leaves of *Eu3* plants under the 100 mM NaCl treatment, implying in an expected H_2_O_2_ increase, which was observed. At the same time, CAT and APX, which catalyze the H_2_O_2_ degradation, and whose activities did not change with the NaCl increase, thus suggesting a failure in detoxifying the excess of H_2_O_2_ produced.

Despite the increased levels of H_2_O_2_ with rising salt concentrations, lipid peroxidation (MDA content) did not follow the same trend, indicating that H_2_O_2_ excess was mitigated by a non-enzymatic antioxidant system. Several compounds have been suggested to reduce ROS in plants under stress, such as ascorbic acid, glutathione, flavonoids, α-tocopherol, and carotenoids (García-Caparrós et al. 2021; Wang et al. 2024).

Considering these hypotheses, based on compounds that were measured, we notice a considerable increase in proline and Trp with rising salt concentrations in both NILs. The increase in proline levels is a common response to salt stress and other types of stress, associated with cellular redox balance, osmotic regulation, ROS scavenging, and stabilization of subcellular structures (Paschalidis et al. 2019; Ghosh et al. 2022).

The aromatic amino acid tryptophan is a precursor of important metabolites, such as auxin (IAA) and melatonin, the latter also being involved in plant defense against abiotic stresses (Li et al. 2019; Khan et al. 2020). Although melatonin levels were not quantified in the present study, previous reports indicate that the availability of Trp directly influences melatonin biosynthesis, especially under stress like salt stress (Li et al. 2019; Hanci and Tuncer 2020). Exogenous application of Trp has been shown to enhance drought stress tolerance in maize by significantly improving key physiological parameters, such as relative water content, membrane stability, and potassium uptake, and Trp also appears to function as an osmolyte and stomatal regulator, contributing to better cell turgor and photosynthetic performance during water deficit (Hanci 2019). Overall, its dual role in modulating hormonal balance and osmotic adjustments suggests that Trp can be an effective agent for improving stress resilience. However, the involvement of specific pathways, including melatonin-related mechanisms, remains hypothetical in the context of this study.

In addition, proline and Trp had higher averages in *eu3-a* plants, and MDA was lower under the 100 mM NaCl treatment compared to *Eu3* plants. A hypothesis is that *eu3-a* plants, already under stress from excess urea, induced higher expression of these genes than *Eu3* plants, and even the urea concentration in *eu3-a* plants was reduced in treatments with added NaCl (Fig. 3A). In the leaves of *eu3-a* plants, the urea concentration was more than 20 times higher than in *Eu3* plants (Fig. 3A). The substantial urea concentration in *eu3-a* plants has been previously observed (Souza et al. 2020a, b). However, in *eu3-a* plants, the urea concentration was higher in the control than 50 mM and 100 mM treatments. The fact that the urea concentration decreased with increasing NaCl levels may be related to the observed increase in free Arg, which was likely functioning as a compatible solute for cellular homeostasis due to saline stress (Khan et al. 2020). But the increase in Arg concentration was only evident in the 100 mM NaCl treatment. Additionally, the increase in free ammonia, observed in salt treatments, could potentially suppress urea production (Urra et al. 2022), since urea hydrolysis by urease releases ammonium when the enzyme is active.

In general, saline stress led to an increase in the concentration of free amino acids, such as proline, Trp, and Arg, as mentioned earlier, as well as Ser and Asn, which could contribute to protein stabilization, osmotic adjustment, and assist in ROS detoxification, thereby supporting cellular homeostasis (Khan et al. 2020). On the other hand, the decrease in Orn and Ctr levels may be related to the increase in Arg, as both are precursors of this amino acid (Kawade et al. 2023).

Concerning polyamines, the reduction in Put concentration observed in both NILs as NaCl concentration increased could be explained by the possible redirection of nitrogen metabolism toward the production of other compounds, such as proline and Trp, predominantly. Moreover, polyamines as Put, are precursors of secondary metabolites, such as hydroxycinnamic acid amides (HCAA), phenolic compounds, and alkaloids (Pál et al. 2021); thus, a reduction in Put levels could be associated with the synthesis of secondary metabolites. Additionally, other metabolites, such as proline and Trp (most probably the downstream product, melatonin), which increase in response to stress, may play a major role in salt stress tolerance in soybean.

Regarding the expression of genes involved in the Arg catabolism pathway, it was observed that most genes were affected by higher NaCl concentrations in both genotypes, particularly in *eu3-a* plants. Considering the metabolite results, despite the increase in expression of all the aforementioned genes, the levels of the corresponding metabolites exhibited a reduction under saline stress, including urea, Orn, Put, and Ctr. We hypothesized that the increase in expression could be due to a decrease in the activity of these enzymes due to stress, which at the same time led to a reduction in these compounds, except in the case of urease, as it is not active in *eu3-a* plants.

In the case of urease, the increase in expression in *eu3-a* plants, may be related to a decrease in nitrogen balance in plants; once the expression of urea-N-responsive genes is altered under low urea/N conditions in higher plants (Souza et al. 2020a, b; Sharma et al. 2023). Therefore, the observed reduction in urea levels under salt stress does not reflect ubiquitous urease (UU) transcript abundance, but may result from impaired urease functionality and broader disruptions in nitrogen metabolism as evidenced by the reduction in nitrate and the increase in ammonium levels.

### Copper-induced photosynthesis reduction: enhanced sensitivity in *eu3-a *plants compared to *Eu3* plants

Unlike the results observed under saline stress, photosynthesis in plants exposed to Cu was generally less affected, with a significant reduction detected only in *eu3-a* plants under the 50 µM Cu treatment. This finding suggests that Cu toxicity may have a more pronounced effect on *eu3-a* plants, potentially due to their altered nitrogen metabolism influencing chlorophyll content. Cu stress is commonly associated with reduced levels of photosynthetic pigments (Wang et al. 2024), thereby negatively affecting photosynthetic efficiency. Although chlorophyll content was not evaluated in the present study, we observed a decrease in nitrate levels in both genotypes, indicating that Cu broadly impaired nitrogen availability.

Excess Cu has been reported to reduce nitrogen (N) content in plants by inhibiting key enzymes involved in nitrogen assimilation, particularly NR. This inhibition may result from the binding of Cu^2^⁺ ions to sulfhydryl (–SH) groups within the NR enzyme, leading to its inactivation (Xiong et al. 2006; Hussain et al. 2020). Moreover, Cu can antagonize molybdenum (Mo)—a critical cofactor for NR—further compromising nitrate reduction and exacerbating nitrogen imbalance (Kabata-Pendias and Pendias 2001; Li and Keller 2023). In addition, *eu3-a* plants accumulated substantially higher levels of urea, and the nitrogen contained in this compound remains largely unavailable for metabolic use. This suggests that *eu3-a* plants may be more susceptible to Cu-induced disruptions in nitrogen assimilation.

As Cu concentrations increased, both NILs exhibited elevated ammonium levels, a response similar to that observed under salt stress. This accumulation may be associated with enhanced photorespiration; however, in contrast to salt stress, no increase in Ser content was detected under copper stress, suggesting that photo-respiratory flux was not markedly stimulated. Additionally, ammonium accumulation may result from stress-induced limitations in key nitrogen-assimilating enzymes, particularly GS and GOGAT (Xie et al. 2025).

These enzymes are central to ammonium incorporation into amino acids, and reduced GS activity is often linked to the accumulation of free ammonium under adverse conditions (Xie et al. 2025). Supporting this hypothesis, Güleryüz et al. (2015) observed reduced GS activity in *Verbascum olympicum*, a Cu-tolerant species, under copper stress, although ammonium content was not assessed.

Under 50 µM Cu, ammonium accumulation was more pronounced in *Eu3* plants, which appeared more sensitive and less efficient in regulating free ammonium levels. In contrast, ammonium accumulation was lower in *eu3-a* plants, likely due to the absence of urease activity, which led to urea retention rather than its conversion into ammonium.

Furthermore, under 10 µM Cu, *Eu3* plants exhibited increased urease gene expression, a pattern also observed under saline conditions. This upregulation may represent a compensatory mechanism in response to nitrogen imbalance, such as the observed nitrate depletion, intended to enhance urea hydrolysis and restore nitrogen availability (Polacco and Holland 1993). However, this mechanism may also contribute to the accumulation of free ammonium, which can be toxic if not efficiently assimilated. Interestingly, in our study, total amino acid content remained unchanged under Cu treatments, suggesting that although nitrate and ammonium metabolism were affected, the overall amino acid pool remained stable, despite shifts in the composition of individual free amino acids.

Regarding lipid peroxidation, the MDA concentration remained unchanged across all copper concentrations in both NILs. However, H_2_O_2_ concentration increased notably in *Eu3* plant leaves treated with 50 µM copper and, additionally, there was a decrease in CAT activity, indicating inadequate detoxification of H_2_O_2_ in *Eu3* plants under 50 µM copper stress, thus affecting the cellular redox homeostasis.

In different soybean cultivars subjected to Cu stress in nutrient solution, Schwalbert et al. (2019) observed similar results: MDA levels did not change, whereas H_2_O_2_ levels increased. However, CAT and APX activities were not assessed in their study, and it was suggested that catalase, peroxidases, or other antioxidant enzymes might have contributed to the observed effects. Here, CAT activity decreased in *Eu3* plants, while APX activity remained unchanged in *Eu3* and decreased in *eu3-a* plants. Therefore, these enzymes do not appear to be primarily responsible for controlling MDA levels under Cu stress. Our findings suggest that other antioxidant enzymes or non-enzymatic antioxidant compounds may play a more significant role in mitigating the effects of H_2_O_2_ on lipid peroxidation in these genotypes.

Chen et al. (2015) also observed that MDA content did not change in a hydroponic experiment with Moso bamboo (*Phyllostachys pubescens*) with Cu supplementation, while SOD and POD activities decreased. The authors suggested that the limited translocation of Cu from roots to leaves helped reduce its impact on lipid peroxidation. In our study, Cu concentrations in roots and leaves were not evaluated. However, it is well established that under high Cu conditions, plants tend to immobilize Cu in the root system, and the translocation rate to the shoots decreases with increasing Cu stress (Wang et al. 2024). This mechanism contributes to reducing Cu-induced toxicity in the aerial parts of the plant (Chen et al. 2015; Wang et al. 2024). Therefore, it is plausible that a similar process occurred in our experimental conditions.

Thounaojam et al. (2012) observed in rice under Cu excess, an increase of guaiacol peroxidase (GPX) and ascorbate peroxidase (APX) activities. The authors suggested that these enzymes were effective in mitigating H_2_O_2_ and reported increases in ascorbate, glutathione, and proline levels; however, MDA content still increased.

Concerning the analyzed genes, the majority showed higher expression levels in *Eu3* compared to *eu3-a* with increasing copper concentration. Despite the upregulation of genes *OAT* (at 50 µM) and *P5CR-1* (at 10 µM), there was no corresponding increase in proline levels, which remained consistent between NILs and across Cu concentrations in *Eu3*.

Although *ADC-1* and -*2* (arginine decarboxylase genes), the agmatine deiminase gene *AGD* (agmatine deiminase gene), *CPA-1*, -*2*, -*3*, and *ARGAH-2,−3* (arginase genes) showed increased expression in *Eu3* plants compared to *eu3-a* plants at 10 µM of Cu, no significant increase in Put was observed, with only a slight increase in Spm. Interestingly, the Put concentration in the control treatment of *eu3-a* was higher than that observed in *Eu3* plants, suggesting elevated Put levels in the mutant. It is hypothesized that the increased gene expression could be attributed to feedback regulation due to a decrease in enzyme activity. Unlike salt stress, no reduction in metabolite levels was observed under copper stress; their levels remained unchanged.

From an agronomic perspective, salinity is a major constraint on soybean productivity, adversely affecting growth, development, and yield (Cheng et al. 2024). Although soybean is generally classified as moderately salt-tolerant, it is notably sensitive to NaCl concentrations between 50 and 100 mM, which are sufficient to induce physiological, biochemical, and molecular disturbances and to discriminate between tolerant and sensitive genotypes (Kokebie et al. 2024). Copper (Cu) stress is also an important concern due to the increasing use of Cu-based fungicides and environmental contamination; soybean tends to be more sensitive to excess Cu than crops, such as maize and common bean, but less sensitive than rice, with substantial variability among cultivars (Engelhardt et al. 2020).

In this context, our results underscore the importance of nitrogen metabolism and antioxidant strategies in plant tolerance to both salinity and copper excess. The contrasting responses between *Eu3* and *eu3-a* plants indicate that the regulation of nitrate, ammonium, and urea pools, together with the capacity to mitigate oxidative stress, are key determinants of stress resilience. These findings provide physiological and biochemical indicators that may support the selection of more tolerant genotypes and inform crop management strategies in environments affected by salinity or metal toxicity.

Some limitations should be considered when interpreting our results. Stress was applied for a relatively short period (5 days), and analyses were restricted to leaf tissues. In addition, a more comprehensive evaluation of nitrogen metabolism, including key enzymes, such as NR, GS, and GOGAT, as well as other antioxidant compounds (e.g., glutathione, flavonoids, and melatonin), was beyond the scope of this study. Future investigations incorporating these analyses, together with pigment content and metal quantification (in the case of copper stress), may provide a more complete understanding of the observed responses.

## Conclusion

The *eu3-a* mutant displayed higher tolerance to salt stress but was more sensitive to copper stress. Salt stress significantly affected photosynthetic parameters in both NILs, while *eu3-a* plants showed better preparation for stress due to urea accumulation and higher expression of genes in the arginine–urea metabolism pathway. Antioxidant mechanisms were less impacted in *eu3-a* plants under salt stress. Both stresses impaired nitrogen metabolism, increasing ammonia levels and reducing nitrate concentration. Further research is needed to clarify the mechanisms underlying *eu3-a*'s response to both excess Cu and salt stress, with particular focus on enzyme activities related to urease-related nitrogen metabolism and the potential contribution of non-enzymatic antioxidants, such as secondary metabolites, glutathione, and ascorbate. Moreover, under salt stress, studies involving both genotypes are essential to elucidate the factors driving the increased Trp content and to determine the role of Trp or its metabolic derivatives in mitigating salt-induced stress.

## Supplementary Information

Below is the link to the electronic supplementary material.

Supplementary file1 (DOCX 2323 KB)

Supplementary file2 (DOCX 2322 KB)

## Data Availability

The datasets generated and analyzed during the current study are available in the Zenodo repository, 10.5281/zenodo.17204324.

## Abbreviations

APX: Ascorbate peroxidase
CPA: *N*-Carbamoylputrescine amidohydrolase
*Eu3*: Dominant homozygous
*eu3-a*: Urease-null activity (formerly, *eu3-e1*)
GOGAT: Glutamate synthase
GS: Glutamine synthetase
MDA: Malondialdehyde
NILs: Near-isogenic lines (*Eu3* and *eu3-a*)
NOS-like: Nitric oxide synthase like
NR: Nitrate reductase
OAT: Ornithine aminotransferase
P5CR: Pyrroline-5-carboxylate reductase
Trp: Tryptophan
