# Supplementary material for: Urease-null soybean (eu3-a) under salt and copper stress: nitrogen metabolism, antioxidant defense, and arginine pathway genes
Source: Planta. 2026 Apr 16;263(5):133. doi: 10.1007/s00425-026-05001-2 (PMC13086885; doi:10.1007/s00425-026-05001-2)
Supplement: Supplementary file 1 — Supplementary file1 (DOCX 2323 KB) [file 425_2026_5001_MOESM1_ESM.docx]

**Supplementary material 1**

**Tab S1.** Phytozome IDs for *Glycine max* Wm82.a2.v1 genes and the forward and reverse primers for key enzymes involved in the arginine degradation pathway.

| **Enzymes** | **Abbreviation** | **ID Phytozome** | **Primer F** | **Primer R** |
| --- | --- | --- | --- | --- |
| Agmatina deiminase | AGD | Glyma.17G083400 | GTGCCAGGGAGATTGTTTTG | GCTCAAATGGCTGGTTGTTG |
| Arginase | ARG-2 | Glyma.17G131300 | CCTGCACAATCTTCAAGGTG | ATCTTTGCAGCCAGTTCTCG |
|  | ARG-3 | Glyma.01G140200 | TGAGAGAACTCGCTGCAAAG | AAATTGAAAAGTTGAGTGCATTG |
| Arginine decarboxylase | ADC-1 | Glyma.04G007700 | GGAAGGGTTGTGTGAATTGG | GGGATAATGGGAAACACCTG |
|  | ADC-2 | Glyma.06G007500 | TCTGTTTTCACGTCCGTTCC | TGTCACAAGTCAAGTCCGAGAG |
| N-carbamoyl-putrescine amidohydrolase | CPA-1 | Glyma.08G074800 | TGCTGACGATAAAGACGAAGC | TAGATCTGGACGCCTATCACG |
|  | CPA-2 | Glyma.12G185700 | GCAGGACCTACAGGAGAAATTG | ATAGATCAGGACGCCTATCACG |
|  | CPA-3 | Glyma.13G315800 | GCAGGACCTACTGGAGAAATTG | GCAATGCCTCATGGATTTG |
| Nitric oxide synthase | NOS-1 | Glyma.11G101800 | TGGGCATCTCCTTATCTTCC | CATGGAATTCCCTTCTCACC |
| Ornithine aminotransferase | OAT-1 | Glyma.05G141900 | TGTAGGAGTTAAGGGCTCATTTG | GGCATGGCAAATCTAGAGAAC |
|  | OAT-2 | Glyma.08G097800 | TATTGGGGTTGCCACAGAAG | TGGATCCCAAACAGAGGTTC |
| Pyrroline-5-carboxylate reductase | P5CR-1 | Glyma.03G129100 | CGTTTTCTCGGTCAAACCTC | AGCGACCGAAACCAAAAG |
|  | P5CR-2 | Glyma.19G131500 | TCACCGTTCTCCCTTCAAAC | TTCGACACCACGTCTTTCAC |
| Ubiquitous urease | UU | Glyma.11G248700 | ATCAAAGGTGGTGAGGTTGC | ACTACCAGCCTTGCCAAATG |
| Reference genes* | CYP2 | Glyma.12G024700 | CGGGACCAGTGTGCTTCTTCA | CCCCTCCACTACAAAGGCTCG |
|  | ELFI B | Glyma.02G276600 | GTTGAAAAGCCAGGGGACA | TCTTACCCCTTGAGCGTGG |
|  | ACTI I | Glyma.18G290800 | CGGTGGTTCTATCTTGGCATC | GTCTTTCGCTTCAATAACCCTA |
