# Supplementary material for: Urease-null soybean (eu3-a) under salt and copper stress: nitrogen metabolism, antioxidant defense, and arginine pathway genes
Source: Planta. 2026 Apr 16;263(5):133. doi: 10.1007/s00425-026-05001-2 (PMC13086885; doi:10.1007/s00425-026-05001-2)
Supplement: Supplementary file 2 — Supplementary file2 (DOCX 2322 KB) [file 425_2026_5001_MOESM2_ESM.docx]

**Supplementary material 2**

**Tab S2.** Biometric measurements of *Eu3* and e*u3-a* plants (NILs) with increasing Cu concentrations (0, 10, and 50 µM). Measurements were made at the end of the experiment (5 days).

| **NILs** | **Cu (µM)** | **LA (cm^2^)** | **LFM (g)** | **RFM (g)** | **SFM (g)** | **LDM (g)** | **RDM (g)** | **SDM (g)** |
| --- | --- | --- | --- | --- | --- | --- | --- | --- |
| ***Eu3*** | **0** | 359.7 ± 35.5 | 5.8 ± 0.7 | 6.4 ± 0.7 | 5.2 ± 0.5 | 1.2 ± 0.12 | 0.5 ± 0.09 | 0.8 ± 0.12 |
|  | **10** | 329.0 ± 66.9 | 5.1 ± 1.2 | 4.6 ± 1.2 | 4.5 ± 1.2 | 1.1 ± 0.25 | 0.4 ± 0.09 | 0.8 ± 0.19 |
|  | **50** | 385.0 ± 15.1 | 6.1 ± 0.3 | 4.6 ± 0.3 | 5.3 ± 0.4 | 1.4 ± 0.07 | 0.5 ± 0.01 | 1.1 ± 0.09 |
|  |  |  |  |  |  |  |  |  |
| ***eu3-a*** | **0** | 367.9 ± 50.6 | 5.9 ± 0.8 | 5.8 ± 0.6 | 5.7 ± 0.9 | 1.3 ± 0.19 | 0.5 ± 0.06 | 1.0 ± 0.17 |
|  | **10** | 381.8 ± 27.5 | 5.3 ± 0.6 | 5.0 ± 1.1 | 5.4 ± 0.8 | 1.1 ± 0.06 | 0.4 ± 0.07 | 0.9 ± 0.12 |
|  | **50** | 349.0 ± 52.4 | 4.7 ± 0.7 | 4.0 ± 0.9 | 4.7 ± 0.5 | 1.1 ± 0.17 | 0.4 ± 0.09 | 1.0 ± 0.09 |
|  |  |  |  |  |  |  |  |  |

Data represents mean values (n=5) ± standard error. The absence of letters statistical suggests no statistical difference in the comparison among NILs within each treatment or between treatments within each NILs. (Tukey test, P < 0.05). Abbreviations: LA= leaf area; LFM= leaf fresh mass; RFM= root fresh mass; SFM= stem fresh mass; LDM=leaf dry mass; RDM=root dry mass; SDM=stem dry mass.
